# Supplementary material for: MetaRibo-Seq measures translation in microbiomes
Source: Nat Commun. 2020 Jun 29;11:3268. doi: 10.1038/s41467-020-17081-z (PMC7324362; doi:10.1038/s41467-020-17081-z)
Supplement: Supplementary file 10 — Supplementary Data 7 [file 41467_2020_17081_MOESM10_ESM.zip › File2/Confidence_VeryHigh_Taxonomy/200988_out.krona.html]

Javascript must be enabled to view this page.

members
magnitude
magnitudeUnassigned
count
unassigned
taxon
rank

200988\_out

8

2
8
superkingdom

2
phylum
1224

2
class
28211

356
1
order

SRS097889\_contig\_number\_10907

order
1
204457

41297
family
1

genus
1
1960290


SRS058070\_contig\_number\_contig-100\_4189.118707
species
1
1234595

1239
6
phylum

1263003
1
species

SRS101376\_contig\_number\_16083

class
4
186801

order
4
186802

541000
1
family

1
genus
946234

292800
species
1

SRS013098\_contig\_number\_43783

3
family
216572

genus
3
459786

1897011
species
3

SRS049896\_contig\_number\_27623SRS142503\_contig\_number\_47653SRS143070\_contig\_number\_6215

1262992

SRS043841\_contig\_number\_contig-100\_941.942
species
1
